# Association of serum lipids with level of blood pressure in type 2 diabetic patients

**DOI:** 10.12861/jrip.2014.15

**Published:** 2013-11-03

**Authors:** Hamid Nasri, Saeed Behradmanesh, Ali Ahmadi, Azar Baradaran, Parto Nasri, Mahmoud Rafieian-Kopaei

**Affiliations:** ^1^Department of Nephrology, Division of Nephropathology, Isfahan University of Medical Sciences, Isfahan, Iran; ^2^Medical Plants Research Center, Shahrekord University of Medical Sciences, Sharekord, Iran; ^3^Department of Epidemiology, Shahid Beheshti University of Medical Sciences, Tehran, Iran; ^4^Department of Pathology, Isfahan University of Medical Sciences, Isfahan, Iran

**Keywords:** Hypertension, Dyslipidemia, Diabetes

## Abstract

**Introduction:** Dyslipidemia and high blood pressure in diabetic patients increase the risk of microvascular and macrovascular complications.

**Objectives:** This study was conducted to investigate the association between serum lipids and level of blood pressure in type 2 diabetic patients (T2D).

**Patients and Methods:** A prospective analytical study was carried out in 60 patients with T2D of both genders. None of the patients had a history of hypertension, and none was treated with antihypertensive drugs. Resting systolic blood pressures and fifth phase diastolic blood pressures were measured three times while the subjects were seated, and the results were averaged second and third measurements. Sixty patients with T2D were enrolled to the study. None of the patients who had a history of gout, was treated with allopurinol or treated with antihypertensive drugs previously.

**Results:** Of 60 participants, mean of serum creatinine was 0.98±0.22 mg/dL. Mean of systolic and diastolic blood pressure was 133±13 mmHg and 84±7.4 mmHg respectively. In this study, a significant positive correlation of serum cholesterol with systolic (r=0.598, p=0.001) and diastolic blood pressure (r=0.584, p=0.001) was seen. Also the associations of serum LDL-C with systolic and diastolic blood pressure were as follow (r = 0.335, p<0.001) and (0.491, p<0.001) respectively. Associations of HDL-C with systolic and diastolic blood pressure were not significance as follow -0.05 and 0.04 respectively.

**Conclusion:** The results of this study suggest that serum cholesterol has a strong association with levels of systolic and diastolic blood pressure in T2D patients. More attention to serum lipids and treatment of dyslipidemia could halt the progress of diabetic kidney disease.

**Implication for health policy/practice/research/medical education:**

A study was conducted, to investigate the association between serum lipids level and level of blood pressure in type 2 diabetes. Sixty patients with T2D was enrolled to the study. The results suggest that serum chlesterol and LDL-C have a significant association with levels of systolic and diastolic blood pressure in type 2 diabetes patients. More attention to the serum lipid levels and treatment of dyslipidemia could halt the progress of diabetic kidney disease.

## Introduction

Dyslipidemia and high blood pressure in diabetic patients increase the risk of microvascular and macrovascular complications (1). Also disturbances of lipid metabolism associated to insulin resistance may be the primary event in the development of type 2 diabetes (1,2). The prevalence of hypertension is higher in diabetic patients than in nondiabetic individuals (3). Hypertension considerably increases the risk of nephropathy (3,4). Hypertension and dyslipidemia are often associated with insulin resistance and aggravation of diabetic kidney disease (3,4). Recently the close association of blood cholesterol and L-DL-C, with essential hypertension have shown (3,4), however limited studies have been conducted on the association of blood pressure with the dyslipidemia of type II diabetes patients.

## Objectives

The aim of this prospective, observational study was to determine whether baseline serum lipid levels are associated with level of blood pressure in T2D.

## Patients and Methods

### Patients

A prospective analytical study was carried out in 60 patients with T2D of both genders. None of the patients had a history of hypertension and none was treated with antihypertensive drugs. Resting systolic blood pressures and fifth phase diastolic blood pressures were measured three times while the subjects were seated, and the results were averaged second and third measurements (5). Hypertension was defined as a blood pressure ≥140/90  mmHg or participant receiving current antihypertensive treatment (3,5).

### Laboratory methods

Venous blood samples were obtained in the fasting state for determinations of serum creatinine, uric acid and hemoglobin A_1_c (HbA_1c_) (reference range 4–6%). 24 hours urine proteinuria was measured too.

### Ethical issues

(1) The research followed the tenets of the Declaration of Helsinki; (2) informed consent was obtained; (3) the research was approved by ethical committee of Shahrekord University of Medical Sciences.

### Statistical analysis

Results were expressed as mean (SD) and were considered as statistically significant when p<0.05. Independent-student’s *t*-test was used for comparison of variables between male and female subjects. Spearman’s rho coefficient correlation for evaluating relations among variables. For association of serum lipids with levels of blood pressure the partial correlation test with adjustment for age, duration of diabetes and serum creatinine was used.

## Results

**Figure 1:**
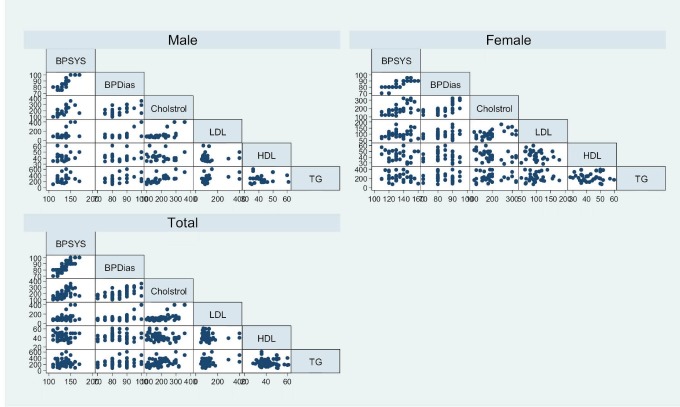
Correlation between systolic blood pressure (BPSYS), diastolic blood pressure (BPDIAS) and cholesterol, LDL-C, HDL-C and TG in male and female patients.

Of 60 participants, 56.7% were female. Mean of age was 57 (±8.3) years. Mean of diabetes duration was 9.2 (±4.9) years. Mean of serum creatinine was 0.98 (±0.22) mg/dL. Mean of systolic and diastolic blood pressure was 133 (±13) mmHg and 84± (7.4) mmHg respectively. Table 1  shows the imortant data of the patients. In this study, there was no significant difference of serum HbA_1c_ and creatinine, between males and females (p>0.05). Similarly, there was no significant difference of proteinuria and levels of systolic or diastolic pressure between males and females (p>0.05). In this study, a significant positive correlation of serum cholesterol with systolic (r=0.598, p=0.001) and diastolic blood pressure (r=0.584, p=0.001) was seen. Also the association of serum LDL-C with systolic and diastolic blood pressure was (r= 0.335, p<0.001) and (0.491, p<0.001) respectively. Associations of HDL-C with systolic and diastolic blood pressure were not significance (-0.05 and 0.04 respectively), however, there were negative. Statistical analysis regarding the associations of serum cholesterol and LDL-C with systolic and diastolic blood pressure per sex are shown in Table 2  and Figure 1 .

**Table 1:** Mean (SD) of systolic (BPSYS), diastolic (BPDIAS) blood pressure, serum cholesterol, LDL-C , HDL-C and TG in male and female subjects

**Variable**	**Male**	**Female**	**P Value**
BPSYS	133.26 (13.7)	133.23 (13.2)	0.99
BPDIAS	84.61 (7.8)	83.67 (7.2)	0.632
CHOLESTROL	196.9 (70)	191.6 (58)	0.747
LDL-C	122.9 (94)	99 (30)	0.179
HDL-C	41.7 (7)	42.5 (8)	0.711
TG	279 (124)	220 (88)	0.032*

*P Value <0.05 was significance

**Table 2:** Correlation and partial correlation between systolic blood pressure (BPSYS), diastolic blood pressure (BPDIAS) and serum cholesterol, LDL-C, HDL-C and TG in male and females and comparison them

**Variable**		**Cholesterol**	**LDL-C**	**HDL-C**	**TG-C**	**Partial correlation**
Male	BPSYS	0.594*	0.472*	0.149	0.249	0.502*
	BPDIAS	0.579*	0.67*	0.2	0.37	0.272
Female	BPSYS	0.6*	0.16	-0.19	0.15	0.61*
	BPDIAS	0.58*	0.22	-0.06	0.02	0.6*
total	BPSYS	0.598*	0.335*	-0.05	0.05	0.565*
	BPDIAS	0.584*	0.491*	0.04	0.197	0.473*

*P<0.05 and significance

## Discussion

In the present study, we found significant positive correlation of serum cholesterol and LDL-C with levels of systolic and diastolic blood pressure. In fact in addition to being associated of hypertension with increasing the risk of cardiovascular disease, dyslipidemia is also associated with an increased risk of kidney damage in patients with type 2 diabetes (7). Chronic kidney disease can increase the risk of cardiovascular disease too (4-6,8). Indeed, few attempts have been paid to analyze the relation between levels of blood pressure and dyslipidemia in individuals with T2DM. A relevant understanding of the interrelation between level of blood pressure and serum lipid levels may be through the understanding of how hypertension is related to the development of diabetic kidney disease and, thereby, aggravates by dyslipidemia (8,9). In a study on 1859 patients were diagnosed as hypertension, Qiao *et al.* found blood cholesterol and L-DL-C are associated closely with level of blood pressure (10). A recent cross-sectional survey on 32004 patients showed that co-existence of high blood pressure and abnormal glucose metabolism is common in Chinese population (11).

## Conclusion

Hypertension and dyslipidemia both are aggravating factors of diabetic nephropathy, thus more attention to dyslipidemia and appropriate treatment of hypertension could attenuate progression of diabetic kidney disease.

## Authors’ contributions

All authors contributed in design of the research. AH analyzed the data. HN, SB, AB, PH, and AH wrote the manuscript. MRK edited the paper. All authors read and approved the paper.

## Conflict of interests

The authors declared no competing interests.

## Ethical considerations

Ethical issues (including plagiarism, data fabrication, and duplicate publication) have been completely observed by the authors.

## Funding/Support

This paper has been derived from the residential thesis. Also, this study was funded by Shahrekord University of Medical Sciences (grant# 898).****
